# (η^6^-Benzene)­chlorido­[(*S*)-2-(4-isopropyl-4,5-di­hydro­oxazol-2-yl)phenolato]ruthenium(II)

**DOI:** 10.1107/S241431462400720X

**Published:** 2024-07-26

**Authors:** Monsuru T. Kelani, Alfred Muller, Koop Lammertsma

**Affiliations:** a Department of Chemical Sciences, University of Johannesburg, Auckland Park, 2006, Johannesburg, South Africa; Purdue University, USA

**Keywords:** crystal structure, ortho­rhom­bic, ruthenium

## Abstract

The title compound, [Ru(C_12_H_14_NO_2_)Cl(η^6^-C_6_H_6_)], exhibits a half-sandwich tripod stand structure and crystallizes in the ortho­rhom­bic space group *P*2_1_2_1_2_1_. The arene group is η^6^ π-coordinated to the Ru atom with a centroid-to-metal distance of 1.6590 (5) Å, with the (*S*)-2-(4-isopropyl-4,5-di­hydro­oxazol-2-yl)phenolate chelate ligand forming a bite angle of 86.88 (19)° through its N and phenolate O atoms. The pseudo-octa­hedral geometry assumed by the complex is completed by a chloride ligand. The coordination of the optically pure bidentate ligand induces metal centered chirality onto the complex with a Flack parameter of −0.056.

## Abstract 2

The title compound, [Ru(C_12_H_14_NO_2_)Cl(η^6^-C_6_H_6_)], exhibits a half-sandwich tripod stand structure and crystallizes in the ortho­rhom­bic space group *P*2_1_2_1_2_1_. The arene group is η^6^ π-coordinated to the Ru atom with a centroid-to-metal distance of 1.6590 (5) Å, with the (*S*)-2-(4-isopropyl-4,5-di­hydro­oxazol-2-yl)phenolate chelate ligand forming a bite angle of 86.88 (19)° through its N and phenolate O atoms.

## Graphical abstract

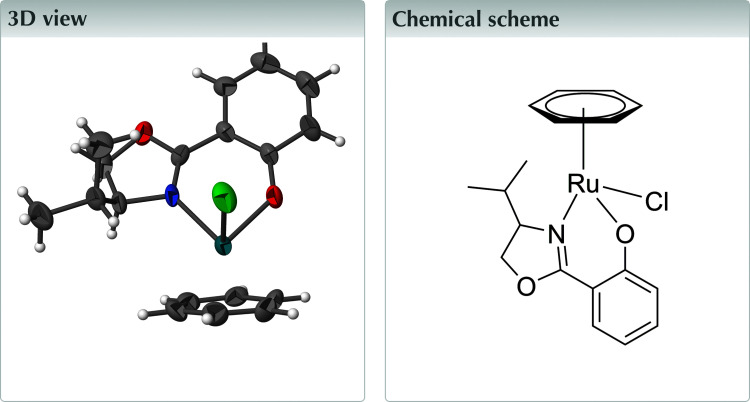

## Structure description

**Figure 1:**
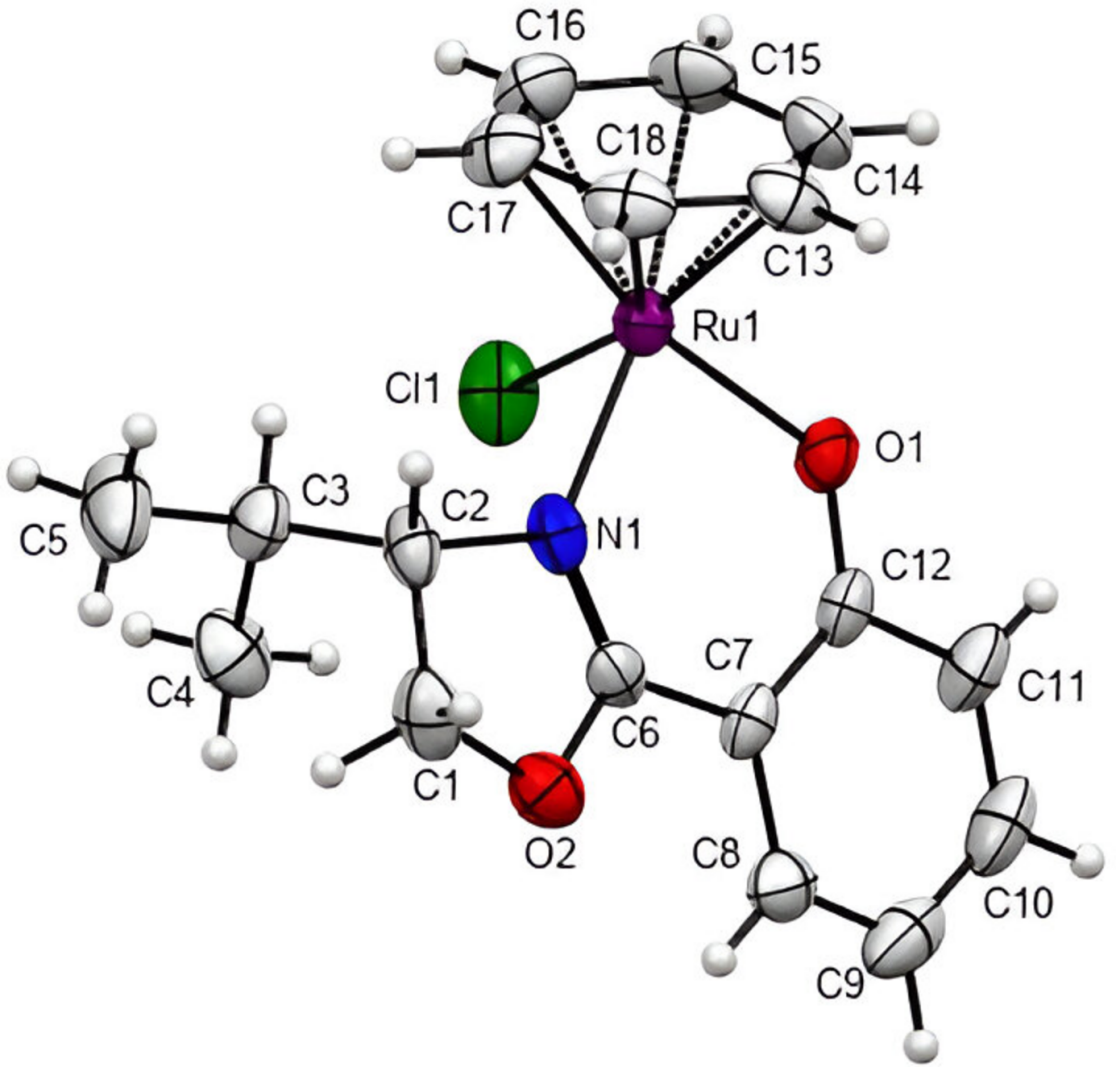
*ORTEP* drawing of the title compound with 50% probability displacement ellipsoids.

Ruthenium complexes have profound applications in various studies relating to chemotherapeutics (Chan *et al.*, 2017 ▸), catalysis (Chavarot *et al.*, 2003 ▸; Hamelin *et al.*, 2007 ▸), electrochemistry (Ryabov *et al.*, 2005 ▸), and photochemistry (Huisman *et al.*, 2016 ▸). The optically pure salicyloxazoline coordinating ligand of the complex is often employed as an auxiliary ligand towards the enantioselective synthesis of chiral-at-metal complexes. The approach relies on the leaving propensity of the benzene and the halo ligands for replacement in the octa­hedral geometry with another achiral ligand system as a strategy in most cases. The choice of the salicyloxazoline ligand is due to its reversible coord­ination upon acid protonation of its phenolate leaving the stereochemistry of the metal complex preserved (Gong *et al.*, 2013 ▸). Thus, the use of the compound is extremely helpful in the synthesis of enanti­omerically pure transition-metal complexes with metal-centred chirality (Gong *et al.*, 2009 ▸, 2010 ▸). The title compound (Fig. 1 ▸) features an optically pure bidentate salicyloxazoline and a chloride ligand within a pseudo-octa­hedral confinement of the three-legged stool while an arene ring occupying the seat of the stool completes the coordination sphere of the ruthenium(II) complex. The bite angle, 86.88 (19)°, of the bidentate ligand is comparable to those of its cymene analogues, 86.68° (Brunner *et al.*, 1998 ▸), 88.29° (Davenport *et al.*, 2004 ▸) and mesitylene analogue, 86.91° (Davenport *et al.*, 2004 ▸) reported in the literature. The Ru forms bond lengths of 2.4176 (19), 2.063 (5) and 2.083 (6) Å to Cl1, O1 and N1, respectively. The crystal packing features weak C—H⋯*X* hydrogen bonding (*X* = O or Cl) in a manner in which each molecular unit is skewed like a satellite dish. Selected torsion angles are given in Table 1 ▸ and details of the hydrogen-bonding geometry in Table 2 ▸.

## Synthesis and crystallization

[*η*^6^-C_6_H_6_)_2_RuCl_2_]_2_ (200 mg, 0.40 mmol, 1 eq), (*S*)-isopropyl-2-(2-hy­droxy­phen­yl)oxazoline (174 mg, 0.84 mmol, 2 eq) and K_2_CO_3_ (122 mg, 0.88 mmol, 2 eq) were dissolved in aceto­nitrile and refluxed for 3 h with continuous stirring. The reaction mixture was cooled to room temperature and then concentrated *in vacuo* under reduced pressure to obtain a single enantiomer of the expected compound. The crude product was purified using column chromatography with silica gel to obtain an orange crystalline compound. Yield, 165 mg (46%, 0.4 mmol). ^1^H NMR (DMSO-*d*_6_) δ 7.24 (*d*, *J* = 7.5 Hz, 1H), 7.05 (*t*, *J* = 7.0 and 7.5 Hz, 1H), 6.62 (*d*, *J* = 8.5 Hz, 1H), 6.28 (*t*, *J* = 7.5 Hz, 1H), 5.71 (*s*, 6H), 4.84 (*d*, *J* = 9.0 Hz, 1H), 4.59 (*dd*, *J* = 3.0 and 8.0 Hz, 1H), 4.41 (*t*, *J* = 9.0 Hz, 1H), 2.56 (*m*, *J* = 6.0 and 7.5 Hz, 1H), 1.0 (*d*, *J* = 7.0 Hz, 3H), 0.68 (*d*, *J* = 6.5 Hz, 3H); ^13^C NMR (DMSO-*d*_6_) δ 164.50, 133.10, 128.57, 128.26, 122.00, 112.40, 108.80, 83.33, 74.71, 67.07, 29.23, 19.12, 14.82; FTIR (neat, cm^−1^) 3067, 1540, 1522, 1489, 1446, 1349, 1255, 1183, 1140, 1069, 826, 763; Elemental analysis calculated for C_18_H_20_ClNO_2_Ru: C, 51.61; H, 4.81; N, 3.34. Found: C, 50.73; H, 4.95; N, 3.64.

## Refinement

Details of the crystal data collection, solution and refinement are provided in Table 3 ▸.

## Supplementary Material

Crystal structure: contains datablock(s) I. DOI: 10.1107/S241431462400720X/zl4075sup1.cif

Structure factors: contains datablock(s) I. DOI: 10.1107/S241431462400720X/zl4075Isup4.hkl

CCDC reference: 2372332

Additional supporting information:  crystallographic information; 3D view; checkCIF report

## Figures and Tables

**Table 1:** Selected torsion angles (°)

O2—C1—C2—N1	−16.4 (7)	C8—C7—C12—O1	179.3 (6)
O2—C1—C2—C3	103.6 (6)	O2—C6—N1—Ru1	174.7 (4)
C6—C7—C8—C9	179.4 (6)	C3—C2—N1—Ru1	71.5 (7)
C10—C11—C12—O1	−179.3 (7)	C7—C6—O2—C1	174.8 (6)

**Table 2:** Hydrogen-bond geometry (Å, °)

*D*—H⋯*A*	*D*—H	H⋯*A*	*D*⋯*A*	*D*—H⋯*A*
C8—H8⋯O2	0.93	2.38	2.725 (9)	102
C17—H17⋯Cl1^i^	0.93	2.80	3.440 (8)	127
C18—H18⋯O1^i^	0.93	2.54	3.405 (8)	156

Symmetry code: (i) 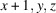.

**Table 3:** Experimental details

Crystal data	
Chemical formula	[Ru(C_12_H_14_NO_2_)Cl(C_6_H_6_)]
*M*_r_	418.87
Crystal system, space group	Orthorhombic, *P*2_1_2_1_2_1_
Temperature (K)	293
*a*, *b*, *c* (Å)	6.5669 (18), 9.414 (3), 27.570 (9)
*V* (Å^3^)	1704.5 (9)
*Z*	4
Radiation type	Mo *K*α
μ (mm^−1^)	1.09
Crystal size (mm)	0.47 × 0.18 × 0.15

Data collection	
Diffractometer	Bruker APEXII CCD
Absorption correction	Multi-scan (*SADABS*; Krause *et al.*, 2015 ▸)
*T*_min_, *T*_max_	0.662, 0.746
No. of measured, independent and observed [*I* > 2σ(*I*)] reflections	9005, 4074, 2937
*R*_int_	0.053
(sin θ/λ)_max_ (Å^−1^)	0.667

Refinement	
*R*[*F*^2^ > 2σ(*F*^2^)], *wR*(*F*^2^), *S*	0.045, 0.089, 0.98
No. of reflections	4074
No. of parameters	210
H-atom treatment	H-atom parameters constrained
Δρ_max_, Δρ_min_ (e Å^−3^)	0.66, −0.41
Absolute structure	Flack *x* determined using 934 quotients [(*I*^+^)−(*I*^−^)]/[(*I*^+^)+(*I*^−^)] (Parsons *et al.*, 2013 ▸)
Absolute structure parameter	−0.05 (6)

Computer programs: *APEX2* and *SAINT* (Bruker, 2010 ▸), *SHELXT* (Sheldrick, 2015*a* ▸), *SHELXL* (Sheldrick, 2015*b* ▸), *Mercury* (Macrae *et al.*., 2020 ▸), *publCIF* (Westrip, 2010 ▸) and *WinGX* (Farrugia, 2012 ▸).

## full crystallographic data

### (η6-Benzene)chlorido[(S)-2-(4-isopropyl-4,5-dihydrooxazol-2-yl)phenolato]ruthenium(II) Crystal data

**Table:**

[Ru(C_12_H_14_NO_2_)Cl(C_6_H_6_)]	*D*_x_ = 1.632 Mg m^−^^3^
*M**_r_* = 418.87	Mo *K*α radiation, λ = 0.71073 Å
Orthorhombic, *P*2_1_2_1_2_1_	Cell parameters from 1424 reflections
*a* = 6.5669 (18) Å	θ = 2.6–22.4°
*b* = 9.414 (3) Å	µ = 1.09 mm^−^^1^
*c* = 27.570 (9) Å	*T* = 293 K
*V* = 1704.5 (9) Å^3^	Plate, orange
*Z* = 4	0.47 × 0.18 × 0.15 mm
*F*(000) = 848	

### (η6-Benzene)chlorido[(S)-2-(4-isopropyl-4,5-dihydrooxazol-2-yl)phenolato]ruthenium(II) Data collection

**Table:**

Bruker APEXII CCD diffractometer	2937 reflections with *I* > 2σ(*I*)
Detector resolution: φ and ω scans pixels mm^-1^	*R*_int_ = 0.053
Bruker APEXII CCD scans	θ_max_ = 28.3°, θ_min_ = 2.3°
Absorption correction: multi-scan (SADABS; Krause *et al.*, 2015)	*h* = −8→6
*T*_min_ = 0.662, *T*_max_ = 0.746	*k* = −12→12
9005 measured reflections	*l* = −36→26
4074 independent reflections	

### (η6-Benzene)chlorido[(S)-2-(4-isopropyl-4,5-dihydrooxazol-2-yl)phenolato]ruthenium(II) Refinement

**Table:**

Refinement on *F*^2^	Secondary atom site location: difference Fourier map
Least-squares matrix: full	Hydrogen site location: inferred from neighbouring sites
*R*[*F*^2^ > 2σ(*F*^2^)] = 0.045	H-atom parameters constrained
*wR*(*F*^2^) = 0.089	*w* = 1/[σ^2^(*F*_o_^2^) + (0.0264*P*)^2^] where *P* = (*F*_o_^2^ + 2*F*_c_^2^)/3
*S* = 0.98	(Δ/σ)_max_ = 0.002
4074 reflections	Δρ_max_ = 0.66 e Å^−^^3^
210 parameters	Δρ_min_ = −0.41 e Å^−^^3^
0 restraints	Absolute structure: Flack *x* determined using 934 quotients [(*I*^+^)-(*I*^-^)]/[(*I*^+^)+(*I*^-^)] (Parsons *et al.*, 2013)
Primary atom site location: dual	Absolute structure parameter: −0.05 (6)

### (η6-Benzene)chlorido[(S)-2-(4-isopropyl-4,5-dihydrooxazol-2-yl)phenolato]ruthenium(II) Special details

**Table:**

Geometry. All esds (except the esd in the dihedral angle between two l.s. planes) are estimated using the full covariance matrix. The cell esds are taken into account individually in the estimation of esds in distances, angles and torsion angles; correlations between esds in cell parameters are only used when they are defined by crystal symmetry. An approximate (isotropic) treatment of cell esds is used for estimating esds involving l.s. planes.
Refinement. The structure solution and refinement were implemented using WinGX software program (Farrugia, 2012). The highest peak and deepest hole are 0.66 and -0.41 e Å^-3^, respectively, which are 1.13 and 0.83 Å away from the ruthenium center. The refinement of the hydrogen atoms was performed isotropically in their idealized geometry while sitting and riding on their anisotropically refined parent atoms with *U*_iso_ = 1.2*U*_eq_ for the aromatic and methine protons, and *U*_iso_ = 1.5*U*_eq_ for the methyl protons.

### (η6-Benzene)chlorido[(S)-2-(4-isopropyl-4,5-dihydrooxazol-2-yl)phenolato]ruthenium(II) Fractional atomic coordinates and isotropic or equivalent isotropic displacement parameters (Å^2^)

**Table:**

	*x*	*y*	*z*	*U*_iso_*/*U*_eq_	
C1	0.2589 (12)	1.2854 (9)	0.8188 (3)	0.047 (2)	
H1A	0.379457	1.320678	0.835165	0.057*	
H1B	0.267789	1.309492	0.784613	0.057*	
C2	0.2375 (11)	1.1261 (8)	0.8256 (2)	0.0362 (17)	
H2	0.364617	1.086811	0.838471	0.043*	
C3	0.1761 (10)	1.0454 (9)	0.7791 (2)	0.0409 (19)	
H3	0.137753	0.948622	0.788452	0.049*	
C4	−0.0048 (18)	1.1122 (8)	0.7531 (2)	0.0508 (17)	
H4A	0.030043	1.206761	0.743036	0.076*	
H4B	−0.119213	1.115928	0.774774	0.076*	
H4C	−0.039225	1.056171	0.725238	0.076*	
C5	0.3629 (12)	1.0355 (12)	0.7454 (3)	0.070 (3)	
H5A	0.333602	0.971968	0.719131	0.105*	
H5B	0.477509	1.000471	0.763443	0.105*	
H5C	0.393965	1.128034	0.732786	0.105*	
C6	−0.0089 (13)	1.2403 (6)	0.86639 (19)	0.0312 (13)	
C7	−0.1874 (9)	1.2786 (7)	0.8950 (2)	0.0316 (16)	
C8	−0.2721 (11)	1.4153 (8)	0.8885 (2)	0.0394 (17)	
H8	−0.212165	1.477639	0.866531	0.047*	
C9	−0.4400 (11)	1.4573 (9)	0.9137 (3)	0.053 (2)	
H9	−0.498466	1.545825	0.908180	0.064*	
C10	−0.5219 (15)	1.3655 (9)	0.9478 (3)	0.056 (2)	
H10	−0.632514	1.395362	0.966250	0.067*	
C11	−0.4456 (10)	1.2333 (9)	0.9552 (3)	0.047 (2)	
H11	−0.506447	1.174317	0.978042	0.057*	
C12	−0.2755 (10)	1.1833 (8)	0.9288 (2)	0.0319 (16)	
C13	0.2044 (11)	0.9128 (8)	0.9641 (2)	0.0424 (19)	
H13	0.238607	0.979015	0.987744	0.051*	
C14	0.0350 (12)	0.8234 (7)	0.9705 (2)	0.042 (2)	
H14	−0.041486	0.830265	0.998837	0.050*	
C15	−0.0200 (14)	0.7245 (7)	0.9352 (2)	0.0456 (18)	
H15	−0.130235	0.664262	0.940233	0.055*	
C16	0.0933 (11)	0.7167 (8)	0.8916 (3)	0.047 (2)	
H16	0.052812	0.654450	0.867215	0.057*	
C17	0.2667 (11)	0.8025 (8)	0.8847 (3)	0.0443 (19)	
H17	0.343741	0.794385	0.856557	0.053*	
C18	0.3229 (10)	0.9013 (8)	0.9212 (3)	0.043 (2)	
H18	0.437009	0.958522	0.916953	0.052*	
N1	0.0747 (7)	1.1175 (6)	0.86312 (18)	0.0311 (14)	
O1	−0.2134 (7)	1.0558 (5)	0.93758 (15)	0.0381 (11)	
O2	0.0756 (7)	1.3444 (5)	0.84022 (17)	0.0456 (14)	
Cl1	−0.2594 (3)	0.8953 (2)	0.84198 (6)	0.0507 (5)	
Ru1	0.00224 (9)	0.93480 (5)	0.90217 (2)	0.02858 (14)	

### (η6-Benzene)chlorido[(S)-2-(4-isopropyl-4,5-dihydrooxazol-2-yl)phenolato]ruthenium(II) Atomic displacement parameters (Å^2^)

**Table:**

	*U*^11^	*U*^22^	*U*^33^	*U*^12^	*U*^13^	*U*^23^
C1	0.044 (5)	0.057 (5)	0.040 (4)	−0.015 (4)	0.012 (4)	0.001 (4)
C2	0.027 (4)	0.054 (5)	0.027 (3)	0.001 (4)	0.008 (3)	0.000 (3)
C3	0.040 (4)	0.047 (5)	0.036 (4)	0.006 (4)	0.010 (3)	−0.003 (4)
C4	0.052 (4)	0.061 (5)	0.039 (3)	−0.005 (8)	−0.013 (4)	−0.001 (3)
C5	0.061 (6)	0.106 (9)	0.044 (5)	0.030 (6)	0.012 (4)	−0.004 (5)
C6	0.041 (4)	0.028 (3)	0.025 (3)	−0.010 (5)	−0.001 (4)	−0.001 (2)
C7	0.027 (4)	0.037 (4)	0.030 (4)	0.000 (3)	0.000 (3)	−0.012 (3)
C8	0.045 (4)	0.038 (4)	0.036 (4)	0.003 (4)	−0.008 (3)	−0.004 (3)
C9	0.054 (6)	0.048 (5)	0.058 (5)	0.017 (4)	−0.015 (4)	−0.021 (4)
C10	0.045 (5)	0.066 (5)	0.055 (4)	0.011 (6)	0.008 (5)	−0.026 (4)
C11	0.043 (6)	0.049 (5)	0.051 (4)	0.002 (4)	0.014 (3)	−0.017 (4)
C12	0.029 (4)	0.038 (4)	0.029 (3)	−0.001 (3)	0.003 (3)	−0.013 (3)
C13	0.040 (4)	0.047 (5)	0.040 (4)	−0.002 (4)	−0.014 (3)	0.008 (4)
C14	0.050 (6)	0.041 (4)	0.035 (3)	−0.003 (4)	0.007 (4)	0.012 (3)
C15	0.046 (5)	0.035 (4)	0.056 (4)	−0.010 (5)	0.008 (5)	0.013 (3)
C16	0.052 (5)	0.035 (4)	0.056 (5)	0.001 (4)	0.001 (4)	−0.003 (4)
C17	0.029 (4)	0.045 (5)	0.059 (5)	0.008 (4)	0.009 (4)	0.001 (4)
C18	0.027 (4)	0.044 (5)	0.059 (5)	0.001 (3)	−0.004 (3)	0.010 (4)
N1	0.019 (3)	0.047 (4)	0.028 (3)	−0.008 (3)	0.005 (2)	0.001 (3)
O1	0.039 (3)	0.037 (3)	0.039 (2)	0.001 (3)	0.017 (2)	−0.002 (2)
O2	0.049 (3)	0.043 (3)	0.046 (3)	−0.007 (2)	0.012 (2)	0.008 (2)
Cl1	0.0276 (10)	0.0772 (16)	0.0475 (10)	−0.0096 (10)	−0.0033 (8)	−0.0106 (10)
Ru1	0.0237 (2)	0.0325 (2)	0.0295 (2)	−0.0020 (4)	0.0034 (3)	−0.0008 (2)

### (η6-Benzene)chlorido[(S)-2-(4-isopropyl-4,5-dihydrooxazol-2-yl)phenolato]ruthenium(II) Geometric parameters (Å, º)

**Table:**

C1—O2	1.452 (8)	C9—C10	1.386 (11)
C1—C2	1.519 (10)	C9—H9	0.9300
C1—H1A	0.9700	C10—C11	1.357 (11)
C1—H1B	0.9700	C10—H10	0.9300
C2—N1	1.490 (7)	C11—C12	1.414 (9)
C2—C3	1.542 (9)	C11—H11	0.9300
C2—H2	0.9800	C12—O1	1.291 (8)
C3—C4	1.523 (12)	C13—C14	1.406 (9)
C3—C5	1.541 (9)	C13—C18	1.420 (9)
C3—H3	0.9800	C13—H13	0.9300
C4—H4A	0.9600	C14—C15	1.396 (9)
C4—H4B	0.9600	C14—H14	0.9300
C4—H4C	0.9600	C15—C16	1.416 (10)
C5—H5A	0.9600	C15—H15	0.9300
C5—H5B	0.9600	C16—C17	1.409 (10)
C5—H5C	0.9600	C16—H16	0.9300
C6—N1	1.282 (8)	C17—C18	1.418 (10)
C6—O2	1.338 (7)	C17—H17	0.9300
C6—C7	1.458 (10)	C18—H18	0.9300
C7—C8	1.413 (9)	N1—Ru1	2.084 (6)
C7—C12	1.418 (9)	O1—Ru1	2.063 (5)
C8—C9	1.363 (9)	Cl1—Ru1	2.4176 (19)
C8—H8	0.9300		
			
O2—C1—C2	104.5 (6)	C8—C9—C10	118.6 (8)
O2—C1—H1A	110.9	C8—C9—H9	120.7
C2—C1—H1A	110.9	C10—C9—H9	120.7
O2—C1—H1B	110.9	C11—C10—C9	122.0 (8)
C2—C1—H1B	110.9	C11—C10—H10	119.0
H1A—C1—H1B	108.9	C9—C10—H10	119.0
N1—C2—C1	101.9 (6)	C10—C11—C12	121.4 (8)
N1—C2—C3	111.3 (5)	C10—C11—H11	119.3
C1—C2—C3	114.1 (6)	C12—C11—H11	119.3
N1—C2—H2	109.8	O1—C12—C11	117.5 (7)
C1—C2—H2	109.8	O1—C12—C7	125.7 (6)
C3—C2—H2	109.8	C11—C12—C7	116.7 (7)
C4—C3—C5	111.3 (6)	C14—C13—C18	119.6 (7)
C4—C3—C2	113.0 (6)	C14—C13—H13	120.2
C5—C3—C2	108.8 (6)	C18—C13—H13	120.2
C4—C3—H3	107.9	C15—C14—C13	121.0 (7)
C5—C3—H3	107.9	C15—C14—H14	119.5
C2—C3—H3	107.9	C13—C14—H14	119.5
C3—C4—H4A	109.5	C14—C15—C16	119.5 (7)
C3—C4—H4B	109.5	C14—C15—H15	120.3
H4A—C4—H4B	109.5	C16—C15—H15	120.3
C3—C4—H4C	109.5	C17—C16—C15	120.6 (7)
H4A—C4—H4C	109.5	C17—C16—H16	119.7
H4B—C4—H4C	109.5	C15—C16—H16	119.7
C3—C5—H5A	109.5	C16—C17—C18	119.5 (7)
C3—C5—H5B	109.5	C16—C17—H17	120.3
H5A—C5—H5B	109.5	C18—C17—H17	120.3
C3—C5—H5C	109.5	C17—C18—C13	119.8 (7)
H5A—C5—H5C	109.5	C17—C18—H18	120.1
H5B—C5—H5C	109.5	C13—C18—H18	120.1
N1—C6—O2	116.4 (7)	C6—N1—C2	107.9 (6)
N1—C6—C7	127.3 (6)	C6—N1—Ru1	127.6 (4)
O2—C6—C7	116.3 (6)	C2—N1—Ru1	124.5 (5)
C8—C7—C12	120.0 (6)	C12—O1—Ru1	129.9 (4)
C8—C7—C6	118.3 (6)	C6—O2—C1	106.5 (6)
C12—C7—C6	121.8 (6)	O1—Ru1—N1	86.9 (2)
C9—C8—C7	121.2 (7)	O1—Ru1—Cl1	85.52 (14)
C9—C8—H8	119.4	N1—Ru1—Cl1	86.26 (15)
C7—C8—H8	119.4		
			
O2—C1—C2—N1	−16.4 (7)	C6—C7—C12—C11	178.7 (6)
O2—C1—C2—C3	103.6 (6)	C18—C13—C14—C15	−0.7 (11)
N1—C2—C3—C4	65.7 (8)	C13—C14—C15—C16	−1.7 (11)
C1—C2—C3—C4	−48.8 (9)	C14—C15—C16—C17	3.3 (11)
N1—C2—C3—C5	−170.2 (6)	C15—C16—C17—C18	−2.5 (11)
C1—C2—C3—C5	75.2 (8)	C16—C17—C18—C13	0.1 (11)
N1—C6—C7—C8	−171.5 (7)	C14—C13—C18—C17	1.5 (11)
O2—C6—C7—C8	7.7 (9)	O2—C6—N1—C2	−5.5 (8)
N1—C6—C7—C12	8.9 (11)	C7—C6—N1—C2	173.7 (6)
O2—C6—C7—C12	−172.0 (6)	O2—C6—N1—Ru1	174.7 (4)
C12—C7—C8—C9	−0.9 (10)	C7—C6—N1—Ru1	−6.2 (10)
C6—C7—C8—C9	179.4 (6)	C1—C2—N1—C6	13.7 (7)
C7—C8—C9—C10	2.9 (11)	C3—C2—N1—C6	−108.3 (7)
C8—C9—C10—C11	−3.0 (13)	C1—C2—N1—Ru1	−166.5 (4)
C9—C10—C11—C12	1.1 (13)	C3—C2—N1—Ru1	71.5 (7)
C10—C11—C12—O1	−179.3 (7)	C11—C12—O1—Ru1	171.7 (4)
C10—C11—C12—C7	0.9 (10)	C7—C12—O1—Ru1	−8.6 (10)
C8—C7—C12—O1	179.3 (6)	N1—C6—O2—C1	−5.9 (8)
C6—C7—C12—O1	−1.1 (10)	C7—C6—O2—C1	174.8 (6)
C8—C7—C12—C11	−1.0 (9)	C2—C1—O2—C6	14.2 (7)

### (η6-Benzene)chlorido[(S)-2-(4-isopropyl-4,5-dihydrooxazol-2-yl)phenolato]ruthenium(II) Hydrogen-bond geometry (Å, º)

**Table:**

*D*—H···*A*	*D*—H	H···*A*	*D*···*A*	*D*—H···*A*
C8—H8···O2	0.93	2.38	2.725 (9)	102
C17—H17···Cl1^i^	0.93	2.80	3.440 (8)	127
C18—H18···O1^i^	0.93	2.54	3.405 (8)	156

Symmetry code: (i) *x*+1, *y*, *z*.

## References

[bb1] Bruker (2010). *APEX2* and *SAINT*. Bruker AXS Inc., Madison, Wisconsin, USA.

[bb2] Brunner, H., Nuber, B. & Prommesberger, M. (1998). *Tetrahedron Asymmetry*, **9**, 3223–3229.

[bb3] Chan, H., Ghrayche, J. B., Wei, J. & Renfrew, A. K. (2017). *Eur. J. Inorg. Chem.* pp. 1679–1686.

[bb4] Chavarot, M., Ménage, S., Hamelin, O., Charnay, F., Pécaut, J. & Fontecave, M. (2003). *Inorg. Chem.* **42**, 4810–4816. 10.1021/ic0341338 12895102

[bb5] Davenport, A. J., Davies, D. L., Fawcett, J. & Russell, D. R. (2004). *Dalton Trans.* pp. 1481–1492. 10.1039/b400747f 15252645

[bb6] Farrugia, L. J. (2012). *J. Appl. Cryst.* **45**, 849–854.

[bb7] Gong, L., Mulcahy, S. P., Devarajan, D., Harms, K., Frenking, G. & Meggers, E. (2010). *Inorg. Chem.* **49**, 7692–7699. 10.1021/ic100229e 20666500

[bb8] Gong, L., Mulcahy, S. P., Harms, K. & Meggers, E. (2009). *J. Am. Chem. Soc.* **131**, 9602–9603. 10.1021/ja9031665 19555100

[bb9] Gong, L., Wenzel, M. & Meggers, E. (2013). *Acc. Chem. Res.* **46**, 2635–2644. 10.1021/ar400083u 23730834

[bb10] Hamelin, O., Rimboud, M., Pécaut, J. & Fontecave, M. (2007). *Inorg. Chem.* **46**, 5354–5360. 10.1021/ic7005502 17530753

[bb11] Huisman, M., White, J. K., Lewalski, V. G., Podgorski, I., Turro, C. & Kodanko, J. J. (2016). *Chem. Commun.* **52**, 12590–12593. 10.1039/c6cc07083c PMC5076553 27711349

[bb12] Krause, L., Herbst-Irmer, R., Sheldrick, G. M. & Stalke, D. (2015). *J. Appl. Cryst.* **48**, 3–10. 10.1107/S1600576714022985 PMC4453166 26089746

[bb13] Macrae, C. F., Sovago, I., Cottrell, S. J., Galek, P. T. A., McCabe, P., Pidcock, E., Platings, M., Shields, G. P., Stevens, J. S., Towler, M. & Wood, P. A. (2020). *J. Appl. Cryst.* **53**, 226–235. 10.1107/S1600576719014092 PMC6998782 32047413

[bb14] Parsons, S., Flack, H. D. & Wagner, T. (2013). *Acta Cryst.* B**69**, 249–259. 10.1107/S2052519213010014 PMC3661305 23719469

[bb15] Ryabov, A. D., Le Lagadec, R., Estevez, H., Toscano, R. A., Hernandez, S., Alexandrova, L., Kurova, V. S., Fischer, A., Sirlin, C. & Pfeffer, M. (2005). *Inorg. Chem.* **44**, 1626–1634. 10.1021/ic048270w 15733006

[bb16] Sheldrick, G. M. (2015*a*). *Acta Cryst.* A**71**, 3–8.

[bb17] Sheldrick, G. M. (2015*b*). *Acta Cryst.* C**71**, 3–8.

[bb18] Westrip, S. P. (2010). *J. Appl. Cryst.* **43**, 920–925.

